# Efficacy of Peak Frequency in Pulmonary Vein Isolation Using Pulsed Field Ablation for Atrial Fibrillation

**DOI:** 10.1111/jce.70187

**Published:** 2025-11-17

**Authors:** Naoki Aizawa, Kaoru Tanno, Takahiro Furuya, Tomoyuki Ishinaga, Keita Shibata, Chisato Sato, Tenjin Nishikura, Naoko Ikeda, Kohei Wakabayashi

**Affiliations:** ^1^ Division of Cardiology Cardiovascular Center, Showa Medical University Koto‐Toyosu Hospital Tokyo Japan

**Keywords:** atrial fibrillation, low‐voltage high frequency, peak frequency, pulsed field ablation

## Abstract

**Introduction:**

Catheter ablation technology for atrial fibrillation (AF) has been improving annually, and pulsed field ablation (PFA) has recently become possible. The optimal protocol has yet to be elucidated, and considering variables, including catheter contact force, is essential for conducting more effective and safer ablation. This study was conducted using peak frequency (PF) mapping to analyze the catheter tip electrode frequency during PFA under the hypothesis that higher PF shows stronger myocardial contact and yields a more pronounced electrical current effect.

**Methods:**

Between December 2024 and April 2025, 12 consecutive patients who underwent PFA for paroxysmal AF using the FARAPULSE system at our hospital were included in this study. Overall, 180 applications were analyzed. The PF before energy delivery was analyzed in the group in which the tip electrode potential disappeared following the first energy delivery using the flower‐type configuration.

**Results:**

The PF of the distal electrode in the group with potential disappearance following the first delivery was significantly higher than it was in the group without disappearance (291 ± 88 Hz vs. 267 ± 70 Hz, *p* = 0.02). When the potential at the tip electrode ranged from 0.5 to 1.5 mV, the area under the curve was 0.73. At a cutoff value of 261 Hz for peak frequency, sensitivity reached 67%, and specificity reached 60%.

**Conclusion:**

Effective PFA may be achieved using the frequency of the catheter tip electrode as an index. Delivering energy to myocardial regions with higher PF may yield greater ablation efficacy.

## Graphical abstract

For sites with potentials ≤ 0.5 mV, the peak frequency value before energy delivery was significantly higher in the potential disappearance group compared to the non‐disappearance group.

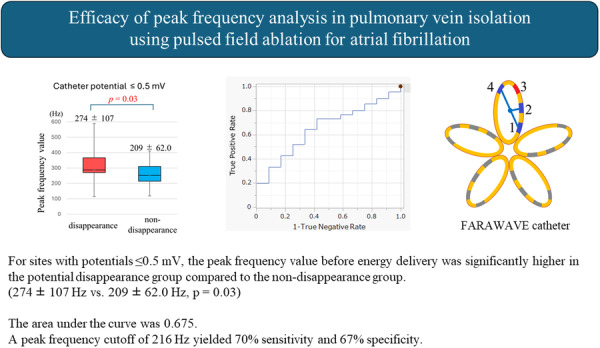

## 1 Introduction

Catheter ablation techniques for atrial fibrillation (AF) are improving annually, and pulsed field ablation (PFA) has recently become feasible. Long‐term outcomes of PFA have shown superiority over conventional thermal ablation in preventing AF recurrence, indicating a potential paradigm shift in the standard treatment for catheter ablation. The effectiveness of PFA has been demonstrated; however, the optimal ablation protocol remains unclear. Conducting more effective and safer ablation requires careful consideration of variables, including catheter contact area and contact force.

The EnSite X EP System (Abbot, Abbott Park, IL, USA), a three‐dimensional (3D) mapping system, has been upgraded to include omnipolar technology near field, enabling calculation of the peak frequency (PF) of acquired potentials. PF mapping in the Ensite X EP system filters potentials at the distal catheter electrode by applying frequency analysis to the intracardiac electrogram. It differentiates electrical activity originating from the myocardium adjacent to the electrode. The PF index shows an estimate of the highest frequency component of the electrogram, with its application to endocardial ventricular mapping being reported [1, 2]. A low PF value may show a far‐field signal. Previous reports suggested that distinguishing near‐field areas characterized by low‐voltage, high‐frequency electrograms from regions with poor catheter contact—showing low‐voltage, low‐frequency electrograms—is possible using PF analysis [3]. In endocardial atrial ablation, a high PF value is assumed to reflect stronger catheter contact with the myocardium. Therefore, this study was conducted using PF mapping with the EnSite X EP System to analyze the catheter tip electrode frequency during PFA under the hypothesis that higher PF shows stronger myocardial contact and yields a more significant electrical current effect.

## 2 Methods

### 2.1 Patient Population

Between December 2024 and April 2025, 12 consecutive patients who underwent PFA for paroxysmal AF at our hospital were included in this study. Patients undergoing a second ablation or deemed ineligible for data analysis were excluded from this study. The relationship between PF and ablation potential was prospectively analyzed.

### 2.2 Ablation Procedure

All patients underwent catheter ablation using the FARAPULSE system. After left atrial access was obtained via the Brockenbrough maneuver under intracardiac ultrasound guidance, electrical localization and frequency data were acquired using ADVISOR HD grid (Abbot, Abbott Park, IL, USA) mapping catheter. Extended pulmonary vein isolation was then conducted on all four pulmonary veins and their antra using a PFA catheter.

**Figure 1:**
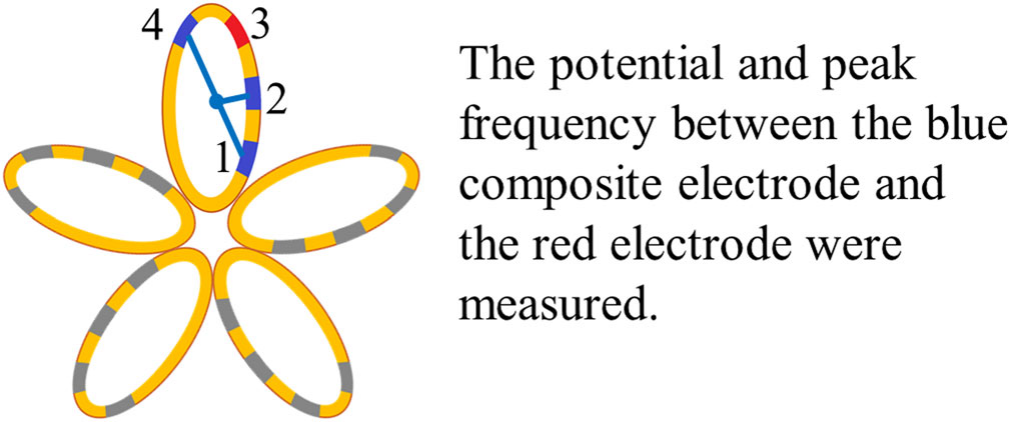
A FARAWAVE catheter with five petals was used to assess the electrical potential and peak frequency between the electrodes on each petal.

**Figure 2:**
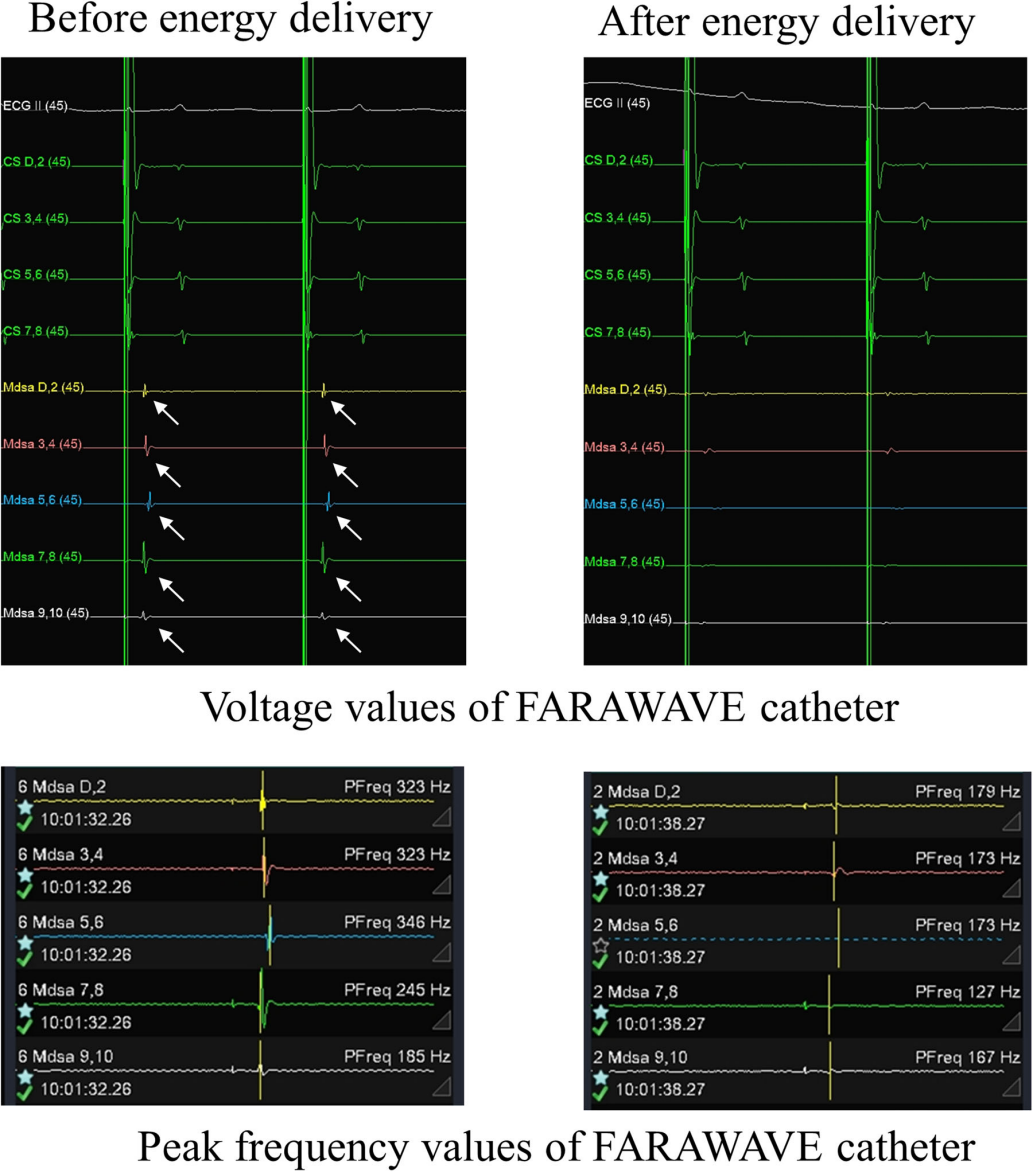
PF values were recorded between electrodes on a single petal of the catheter before and after the first energy delivery. When the potential indicated by the white arrow disappeared after delivery, PF values also decreased.

A FARAWAVE (FARAWAVE, Farapulse Inc., Menlo Park, CA, USA) 12F over‐the‐wire catheter was transformed into a flower configuration and positioned at the ostium of each pulmonary vein. Two consecutive energy deliveries, each using a 2000 V biphasic waveform, were administered. The catheter was then rotated 36 degrees clockwise or counterclockwise around its central axis, followed by two additional energy deliveries. Subsequently, the catheter was reshaped into a pumpkin configuration, and four energy deliveries were delivered in the same fashion as in the flower configuration. Overall, 32 energy deliveries were used, with eight directed to each of the pulmonary veins. Simultaneously, bipolar potentials and PF values were recorded between the electrodes (referred to as tip electrodes) on a single petal of the catheter (Figure 1) before and after the first energy delivery in the flower configuration (Figure 2). Completion of pulmonary vein isolation was confirmed by demonstrating bidirectional block using a mapping catheter to reassess electrical localization in the left atrium.

### 2.3 PF Value Analysis

Electrodes with a pre‐delivery potential of 0.1 mV or less were considered to potentially disappear and were excluded from analysis. Potential disappearance was defined as a post‐delivery electrode potential of 0.1 mV or less following the first energy application. The PF values before energy delivery were compared between the group in which the tip electrode potential disappeared following the first flower‐shaped delivery (potential disappearance group) and the group in which the potential remained (non‐disappearance group). Additionally, a receiver operating characteristic (ROC) curve analysis was used to examine the relationship between PF values and potential disappearance, and the PF cutoff value was calculated.

Subgroup analyses were conducted based on pre‐delivery tip electrode potentials: 0.5 mV or less, 0.5–1.5 mV or less, and 1.5 mV or more.

**Figure 3:**
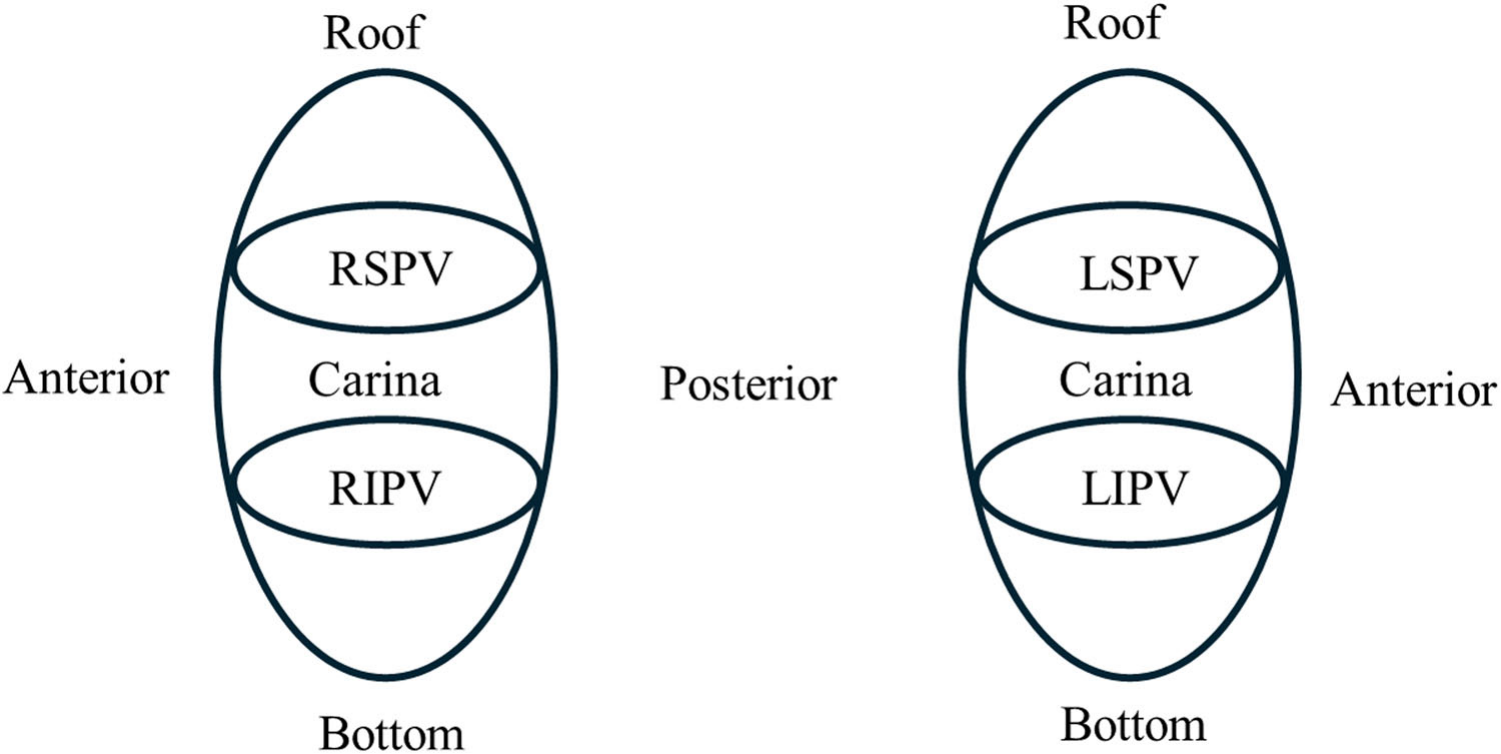
Pulmonary veins were divided into five sections: anterior, roof, posterior, bottom, and carina. The electrical potential and peak frequency were analyzed for each section.

Furthermore, subgroup analyses were conducted based on the location of the pulmonary vein segment to assess the relationship between pre‐delivery PF values and potential disappearance. The four pulmonary veins—right superior pulmonary vein (RSPV), left superior pulmonary vein (LSPV), left inferior pulmonary vein (LIPV), and right inferior pulmonary vein (RIPV)—were divided into five anatomical segments: superior, anterior, inferior, posterior, and carina (Figure 3).

### 2.4 Ablation Device System

Two introducer sheaths and one steerable sheath were inserted through the right femoral vein. A 7F short sheath was inserted through the right internal jugular vein, and a coronary vein catheter was placed. The EnSite X EP System (Abbot, Abbott Park, IL, USA) was used for 3D electroanatomical navigation. The FARAWAVE catheter and a dedicated 13Fr sheath were inserted through the right femoral vein. Intracardiac mapping was conducted using the Advisor HD Grid mapping catheter (Abbot, Abbott Park, IL, USA).

### 2.5 Anesthesia Protocol

General anesthesia (GA) was administered in all cases. Induction of GA was achieved with intravenous fentanyl (1.5–4.3 µg/kg), propofol (0.9–1.3 mg/kg), and rocuronium (0.8–1.0 mg/kg). Airway management was maintained using either tracheal intubation or a supraglottic airway device. Sevoflurane and remifentanil were administered during the ablation procedure.

### 2.6 Outcomes

The primary outcome was the PF value before energy delivery in the disappearance group. The secondary outcome was the incidence of procedural complications, including cardiac tamponade, hemolysis, esophageal injury, and cerebral infarction. Pericardial effusion was monitored to assess complications using intracardiac echocardiography after the procedure. Post‐ablation blood testing was used to evaluate haptoglobin levels and to screen for hemolysis through changes in lactate dehydrogenase (LDH), total bilirubin, and serum potassium levels.

### 2.7 Experimental Ethics and Reporting

This study followed the principles of the Declaration of Helsinki. The Ethics Committee of Showa Medical University (approval number 2025‐0022) approved this study. All patients provided informed consent using an opt‐out approach.

### 2.8 Statistical Analysis

Continuous variables were presented as means ± standard deviation. The potential disappearance and non‐disappearance groups were compared using the parametric *t*‐test for continuous variables and Fisher's exact test for categorical variables. Subgroup analyses were conducted following the location of the pulmonary vein and its anatomical segment. Statistical significance was set at *p* < 0.05. All statistical analyses were conducted using JMP Pro version 17 software (SAS Institute Inc., Cary, NC, USA).

## 3 Results

### 3.1 Patient Background and Baseline Characteristics

Twelve patients underwent PFA for paroxysmal AF. One patient was excluded owing to unsuitability for data analysis, leaving 11 patients for the final analysis.

Overall, 180 energy deliveries were assessed, corresponding to 180 analyzed tip electrode sites. The mean number of energy deliveries per case was 32. Among these, 79 sites were part of the potential disappearance group, whereas 101 sites were assigned to the non‐disappearance group. Table 1 shows the patient background information, including age, sex, body mass index (BMI), comorbidities (hypertension, diabetes, history of heart failure, and stroke), left ventricular ejection fraction, and left atrial volume index, as well as anesthesia time, fluoroscopy time, and left atrial indwelling time of the catheter.

**Table 1:** Patient background and baseline characteristics.

Number of patients	11
Sex	
Male	7 (64)
Female	4 (36)
Average age	71 ± 13
Average BMI	23.1 ± 3.74
Co‐morbid disease	
Heart failure	1 (9.1)
Hypertension	7 (64)
Diabetes	0
Stroke	0
Echocardiography	
LVEF (%)	64 ± 5.8
LAVI	34 ± 7.5
Catheter ablation	
Energy delivery points	32
Anesthesia time (minutes)	106 ± 22.6
Fluoroscopy time (minutes)	22 ± 4.3
Left atrial indwelling time (minutes)	59 ± 23

*Note:* Values are expressed as n or mean ± standard deviation.

Abbreviations: BMI, body mass index; LAVI, left atrial volume index; LVEF, left ventricular ejection fraction.

### 3.2 Primary Outcome

Ablation was successful at all target sites. Pulmonary vein isolation following the first ablation line was achieved in 10 patients.

**Figure 4:**
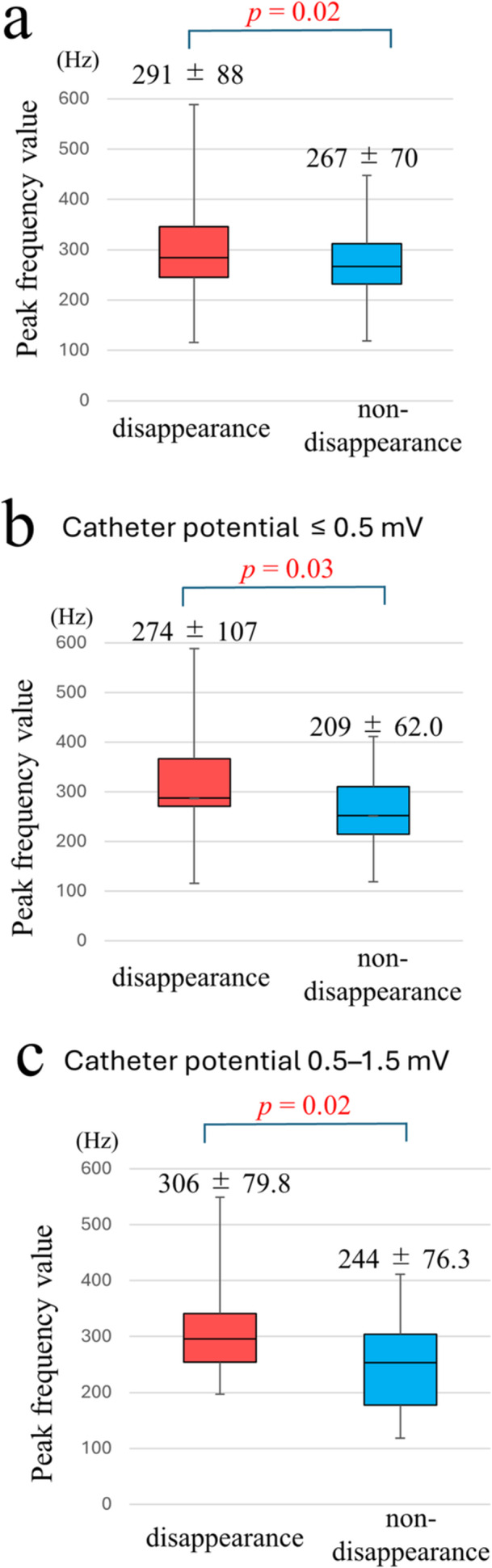
Peak frequency analysis results. A comparison of frequencies at all potentials was shown in (A), (B) showed a comparison of frequencies when the potential at the tip electrode is 0.5 mV or less, and (C) showed frequencies when the potential is 0.5‐1.5 mV.

The PF value before energy delivery was significantly higher in the potential disappearance group than it was in the non‐disappearance group (291 ± 88 Hz vs. 267 ± 70 Hz, *p* = 0.02) (Figure 4a).

In the subgroup analyses, when the potential at the tip electrode before energy delivery was 0.5 mV or less, the PF value in the potential disappearance group was significantly higher than that in the non‐disappearance group (274 ± 107 Hz vs. 209 ± 62.0 Hz, *p *= 0.03) (Figure 4b). A significant difference was also observed when the potential was between 0.5 and 1.5 mV (306 ± 79.8 Hz vs. 244 ± 76.3 Hz, *p* = 0.02) (Figure 4c). Table 2 shows the PF value analysis, as measured by the potential at the tip electrode before energy delivery.

**Table 2:** PF values for each potential before tip electrode energy delivery.

	Potential disappearance		Non‐disappearance		*p* value
	PF value (Hz)	*n*	PF value (Hz)	*n*	
≤ 0.5 mV	274 ± 107	30	209 ± 62.0	12	0.03
0.5–1.5 mV	306 ± 79.8	33	244 ± 76.3	32	0.02
> 1.5 mV	297 ± 61.1	16	293 ± 55.0	57	n.s.

*Note:* Values are expressed as n or mean ± standard deviation.

Abbreviation: PF, peak frequency.

**Figure 5:**
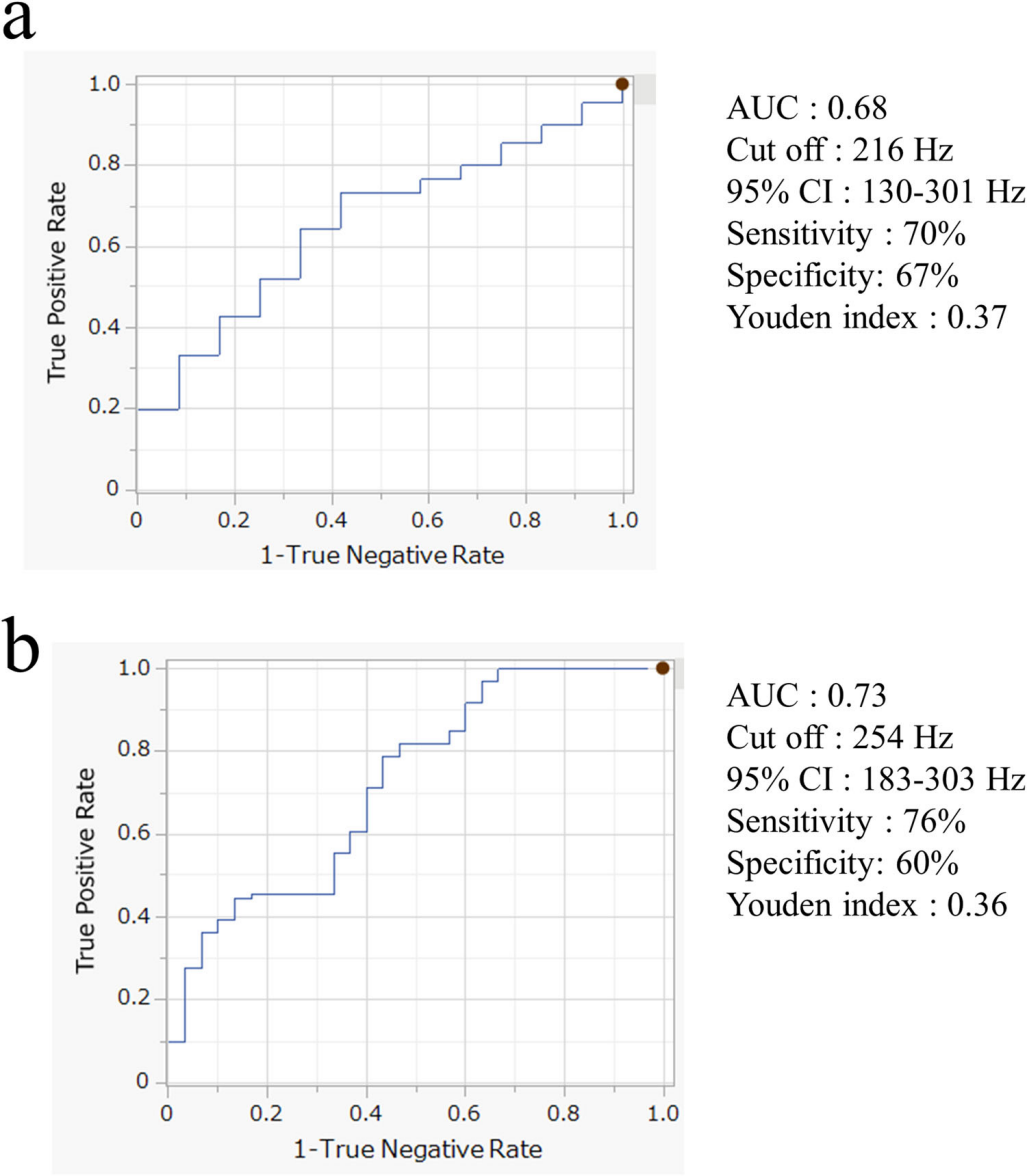
The ROC curve of the relationship between the disappearance of potential and the PF value was shown in figure (A) when the tip potential is 0.5 mV or less and in (B) when it is 0.5‐1.5 mV.

The relationship between the disappearance of potential and the PF value before energy delivery is shown in the ROC curve. For sites with potentials ≤ 0.5 mV, the area under the curve (AUC) was 0.68 (Figure 5a). A PF cutoff of 216 Hz yielded 70% sensitivity and 67% specificity.

For potentials between 0.5 and 1.5 mV, the AUC was highest at 0.73 (Figure 5b). A PF cutoff of 254 Hz provided 76% sensitivity and 60% specificity.

Subgroup analyses based on individual pulmonary veins (RSPV, LSPV, LIPV, and RIPV) revealed no significant differences in PF values before and after energy delivery (Table 3). Similarly, analyses by anatomical segments of the pulmonary veins (superior, anterior, inferior, posterior, and carina) were not significantly different (Table 4).

**Table 3:** PF values for each pulmonary vein.

	Potential disappearance		Non‐disappearance		*p* value
	PF value (Hz)	*n*	PF value (Hz)	*n*	
RSPV	305 ± 97.6	25	284 ± 83.9	22	n.s.
RIPV	285 ± 74.8	19	300 ± 50.8	22	n.s.
LSPV	295 ± 97.6	21	260 ± 61.3	32	n.s.
LIPV	273 ± 78.5	14	235 ± 69.1	25	n.s.

*Note:* Values are expressed as n or mean ± standard deviation.

Abbreviations: LIPV, left inferior pulmonary vein; LSPV, left superior pulmonary vein; PF, peak frequency; RIPV, right inferior pulmonary vein; RSPV, right superior pulmonary vein.

**Table 4:** PF values for pulmonary vein segments.

	Potential disappearance		Non‐disappearance		*p* value
	PF value (Hz)	*n*	PF value (Hz)	*n*	
Anterior	271 ± 86.5	22	241 ± 71.8	32	n.s.
Roof	300 ± 52.1	14	282 ± 65.4	15	n.s.
Posterior	295 ± 84.5	17	289 ± 71.0	25	n.s.
Bottom	318 ± 92.9	6	271 ± 54.8	12	n.s.
Carina	299 ± 114	20	272 ± 69.8	17	n.s.

*Note:* Values are expressed as n or mean ± standard deviation.

Abbreviation: PF, peak frequency.

### 3.3 Secondary Outcome

There were no complications for any of the patients. Post‐procedural measurements showed haptoglobin levels of 52.7 ± 39.8 mg/dL, LDH 226 ± 169 U/L, and total bilirubin 0.58 ± 0.19 mg/dL, with no clinically relevant increases compared to baseline (Table 5).

**Table 5:** Blood test results following ablation.

Number of patients	11
Blood test data	
Hb (g/dL)	11.4 ± 1.54
ΔHb (g/dL)	–2.34 ± 1.23
LDH (U/L)	226 ± 169
ΔLDH (U/L)	−6.78 ± 21.8
Total bilirubin (mg/dL)	0.58 ± 0.19
ΔTotal bilirubin (mg/dL)	−0.10 ± 0.54
Potassium (mEq/L)	4.07 ± 0.26
ΔPotassium (mEq/L)	−0.14 ± 0.27
Haptoglobin (mg/dL)	52.7 ± 39.8

*Note:* Values are expressed as n or mean ± standard deviation.

Abbreviations: Hb, hemoglobin, LDH, lactate dehydrogenase.

## 4 Discussion

In this study, we attempted to use the PF value as a surrogate for contact force. Myocardium with a higher PF value is more likely to show acute effects, indicating that energy exceeding the threshold for irreversible electroporation (IRE) in PFA may be delivered more effectively.

In PFA, membrane permeabilization occurs when a sufficiently strong electric field is applied to cells, leading to increased ion transport and overall membrane instability [4]. Several factors influence membrane permeabilization (pulse amplitude, pulse width, pulse number, biphasic or monophasic waveform, and pulse period length) [5], and membrane permeabilization may be incomplete if the electrode is positioned far from the tissue [6]. Even in tissues with irregular surfaces where close electrode‐tissue contact is less than ideal, PFA potentially creates more uniform lesions compared to those created using radiofrequency (RF) energy [7, 8]; however, electrode‐tissue contact is likely important for optimal results. As an acute effect of PFA, cardiomyocytes exposed to PFA delivery with energies above the IRE threshold lose the ability to contract immediately after the procedure [7].

In clinical studies involving pigs, PFA resulted in an acute block in all lines and showed histological penetration of 97.8%–100% [9, 10]. Non‐randomized studies using the FARAPULSE system showed durable pulmonary vein isolation in 84.8% of cases with a mean energy delivery of 7.2 ± 2.2 per pulmonary vein [11, 12]. Studies using other PFA catheters have also shown 100% acute pulmonary vein isolation [13]; nevertheless, the effect of a single energy delivery was not examined.

This study showed that when conducting PFA using the FARAPULSE system, the potential after the first energy delivery disappeared more significantly when the PF value was 210 Hz or higher, provided the potential before energy delivery was 0.5 mV or less at each tip electrode. When the potential before energy delivery was between 0.5 and 1.5 mV or less, the disappearance of potential was more significant when the PF value was 261 Hz or higher. The PF value increased when the catheter tip made contact with the left atrial wall, suggesting that checking the PF value before delivery may serve as a substitute for assessing catheter tip contact.

Conversely, all cases showed a high first‐pass rate. This suggests that energy delivery was conducted multiple times to the same pulmonary vein; however, the effective application could have been achieved from the second attempt onwards, even if the effect of the first energy delivery was weak. No complications, including hemolysis, occurred as a result of conducting multiple energy deliveries, and the usefulness of the protocol involving multiple energy deliveries appears to remain valid at this time.

The EnSite X EP system, a 3D mapping system, has been upgraded to calculate the PF value of the acquired potentials. PF maps are a novel indicator for slow pathway ablation [14]. EnSite OT Near Field (OTNF) is a proprietary algorithm available on the EnSite X mapping system. Reportedly [1], OTNF automatically tracks the highest frequency signal component along the time course of each intracardiac electrogram, generating a unique PF trace. PF does not necessarily show the highest amplitude component of the electrogram and correlates with the visual clarity of the electrogram morphology, even at low amplitudes.

PF is a new index that quantifies the near‐field component of the electrocardiogram. In normal voltage myocardium, PF values are moderate to high [15]. Conversely, in scarred myocardium, electrical activity is minimized, and PF values are low—less than 150 Hz—suggesting that signals from scarred myocardium may predominantly originate from the far field. However, in low‐voltage myocardium, PF values vary from low to medium to high, and a more heterogeneous structure is observed. Higher PF areas in low‐voltage regions may help identify conduction gaps.

In this study, the ease with which potential disappearance occurred following energy delivery in high PF areas may be owing to the selective blocking of myocardium with the potential to become a conduction gap.

Regarding safety, electroporation presents potential concerns, such as a phenomenon known as arcing [16], which can lead to vapor popping, pericardial effusion, cardiac tamponade, and intramural thrombus. However, no obvious complications were detected in this study. There are concerns regarding potential tissue damage to the phrenic nerve and esophagus—structures adjacent to the myocardium—when PFA electrodes are in close proximity; nonetheless, preclinical evaluations have reported minimal to no damage to adjacent tissues following PFA [8, 17].

This study contributes to PFA by enabling more reliable ablation. Energy delivery guided by PF values may reduce the formation of conduction gaps and allow ablation to be conducted with fewer applications.

### 4.1 Limitations

This study had some limitations. First, the study design was non‐randomized and conducted at a single center, which may have introduced selection bias related to the operator's skill. Second, the number of patients enrolled was very small and the AUC was modest, which may lack evidence of statistical significance. Third, PFA was conducted exclusively under GA, and the effects under deep sedation remained unclear. Fourth, long‐term outcomes were not evaluated; the duration of sinus rhythm maintenance after ablation using PF value as an index and the presence or absence of long‐term complications, including pulmonary vein stenosis, were not evaluated. Finally, PF value measurements were obtained using the flower‐shaped electrode of the FARAWAVE catheter, which had an inter‐electrode spacing of approximately 5 mm. This configuration may have limited the accuracy of near‐field potential detection and PF value measurement compared to mapping catheters, including the HG grid. Future research should evaluate the PF characteristics of other catheters. Moreover, examining the reproducibility of similar effects under deep sedation and conducting a long‐term follow‐up analysis remain essential.

## 5 Conclusions

During PFA, effective ablation appeared achievable using the PF value of the catheter tip electrode as an index. In myocardium with normal potential, there was no correlation between the PF value of the tip electrode and the disappearance of potential after energy delivery. However, a higher ablation effect seemed attainable by targeting high‐frequency sites in relatively low‐potential areas.

## Conflicts of Interest

The authors declare no conflicts of interest.

## Data Availability

The data that support the findings of this study are available from the corresponding author upon reasonable request. Raw data were generated at Showa Medical University Koto‐Toyosu Hospital.
